# Combined effects of resistant dextrin and inulin on the reduction of harmful metabolites during *in vitro* fecal fermentation in older adults

**DOI:** 10.3389/fnut.2026.1909212

**Published:** 2026-09-03

**Authors:** Kazuma Yoshida, Takeshi Matsubara, Shunsuke Morita, Yasuyuki Sakata, Hirofumi Sonoki, Kazuhiro Miyaji

**Affiliations:** Health Care & Nutritional Science Institute, R&D Division, Morinaga Milk Industry Co., Ltd., Zama, Japan

**Keywords:** fecal fermentation, gut microbiota, inulin, metabolomics, older adults, prebiotics, resistant dextrin, trimethylamine

## Abstract

**Background/objectives:**

Aging-related dysbiosis is associated with increased production of harmful gut-derived metabolites. Reducing these metabolites by modulating the gut microbiota is important for maintaining health in older adults. This study aimed to investigate whether a combination of resistant dextrin and inulin suppresses the production of harmful metabolites in the gut microbiota of older adults using *in vitro* fecal fermentation and a metabolomics approach.

**Methods:**

Fecal samples from eight older adults were fermented with resistant dextrin, inulin, their combination, or no saccharides as a control. Potentially harmful metabolites in the fermentation supernatants were assessed by metabolomic analysis. Trimethylamine production was verified by the fecal fermentation with a choline-supplemented medium.

**Results:**

The combination group showed a decreasing trend in the production of deoxycholic acid, kynurenine, and trimethylamine compared with the control group. Assessment of trimethylamine production using a choline-supplemented medium revealed that the combination group had a higher proportion of fecal samples with low trimethylamine production (3 of 8 samples) than either prebiotic-alone group.

**Conclusion:**

Combining resistant dextrin and inulin could reduce the production of some harmful metabolites by the gut microbiota in older adults.

## 1 Introduction

Aging is associated with changes in gut microbiota composition and function (1, 2). These age-related changes may be influenced by factors such as dietary habits, immune function, and lifestyle (3, 4). Such shifts can disturb the balance of the gut microbiota, leading to dysbiosis in older adults. Dysbiosis may contribute to the development and progression of age-related diseases (5). Therefore, maintaining a healthy gut microbiota is particularly important for older adults who are at a higher risk of diseases than younger people.

The gut microbiota influences host health by producing various metabolites (6). Gut microbiota composition and metabolites are influenced by host diet (7, 8). For example, the gut microbiota metabolizes dietary fibers and oligosaccharides to produce short-chain fatty acids, which maintain host immune function (9). However, harmful metabolites are also produced from certain nutrients (e.g., indole from tryptophan and trimethylamine (TMA) from choline and carnitine (10, 11)). In addition, certain harmful metabolites have been implicated in the development of diseases, including cardiovascular disorders and cancer (12, 13). The production of these harmful metabolites is accelerated by dysbiosis resulting from aging and disease (14, 15). Therefore, strategies aimed at modulating the gut microbiota and reducing the production of harmful metabolites are particularly important for promoting the health of older adults.

One of the common methods to improve gut microbiota is taking prebiotics. Prebiotics are defined as “a substrate that is selectively utilized by host microorganisms,” such as fibers (16). The utilization of prebiotics by the gut microbiota varies depending on their structural characteristics, and the metabolites produced from them are different (17, 18). Combining prebiotics with different structures increases the microbiota diversity and changes the amount and composition of metabolites (19, 20). Therefore, ideal prebiotic combinations may exist to reduce the production of harmful metabolites by the gut microbiota.

Metabolomics approaches that comprehensively analyze a large number of metabolites have been used to explore metabolites from the gut microbiota (21, 22). Clinical studies are necessary to analyze the effects of prebiotics on gut microbiota metabolites; however, screening a large number of candidate prebiotic combinations in clinical trials is not feasible. Moreover, clinical studies cannot reveal the changes in the intestinal environment induced by prebiotic intake, such as intestinal pH variation. *In vitro* fecal fermentation can easily simulate the intestinal environment (17). In addition, as the response to prebiotics varies with age, evaluating their effects in *in vitro* fecal fermentation requires fecal samples obtained from older adults. Thus, by combining a metabolomics approach with *in vitro* fecal fermentation using feces from older adults, it is possible to comprehensively investigate the effects of prebiotics on the gut microbiota in older adults.

In a previous study, we demonstrated the usefulness of a combination of two different prebiotics, resistant dextrin (RD) and inulin (Inu), in increasing microbiota diversity and promoting short-chain fatty acid production while reducing gas production during *in vitro* fecal fermentation using samples from older adults (19). We also found that Inu was rapidly utilized by the gut microbiota while RD was consumed more slowly, suggesting that their combination may provide complementary fermentation characteristics and favorably modulate the gut microbial environment. Because harmful metabolite production can be influenced by gut microbiota composition and its metabolic activity, we hypothesized that the combination of RD and Inu may reduce harmful metabolites by improving the overall fermentation environment. Therefore, the present study aimed to comprehensively investigate metabolites and to explore the potential additional benefits of the RD and Inu combination.

This study aimed to use a metabolomics approach to identify harmful metabolites that are expected to decrease during *in vitro* fecal fermentation of samples from older adults following treatment with a combination of RD and Inu. Additional validation studies were conducted by adding substrates for the target metabolites to the fermentation medium.

## 2 Materials and methods

### 2.1 Materials

Resistant dextrin (RD; Fibersol-2) was obtained from Matsutani Chemical Industry Co., Ltd. (Hyogo, Japan). Fibersol-2 is a water-soluble dietary fiber derived from corn starch, consisting mainly of glucose oligosaccharides with DP ≥ 3 and an average molecular weight of approximately 2,000 Da. Fuji FF is a highly purified inulin produced from sucrose with inulin synthase and has a chain length distribution of 3–30, with an average chain length of 14–16. Yeast extract, casitone, and fatty acid (YCFA) medium was prepared as described in Supplementary Table S1.

### 2.2 Fecal sample collection

This study used fecal samples from participants recruited for *in vitro* fecal fermentation studies designed to investigate the relationships among dietary intake, gut microbiota, and metabolites. The same fecal samples were also used in our previous fermentation study (19). Fecal samples were collected from healthy Japanese older adults in accordance with previously described protocols (19). Participants aged 65–75 years living in the Tokyo metropolitan area were recruited through the clinical trial recruitment website “https://www.seikatsu-kojo.jp/ (accessed on 12 June 2026).” The study information and details were posted on the website by the agency operating the recruitment service. Fecal samples were collected in collection tubes and subsequently stored under anaerobic conditions in a household freezer using an AnaeroPouch (Mitsubishi Gas Chemical, Tokyo, Japan). The fecal samples were transported to the Morinaga Milk Industry laboratory within 48 h of collection, under frozen conditions. All samples were stored at −80 °C until use. Thirteen fecal samples were collected. Five fecal samples were excluded before the fermentation experiments. In detail, four samples were excluded because anaerobic conditions could not be maintained during fecal storage. The remaining sample was excluded to ensure the safety of the experimenters because adenovirus was detected. Therefore, eight fecal samples were used in this study. No fecal donors were taking antibiotics or other medications that could affect the gut microbiota. The characteristics of the fecal donors are summarized in Supplementary Table S2. This study was approved by the Ethics Committee of the Japan Conference of Clinical Research (Tokyo, Japan; protocol code: NFSFA-01; date of approval: 18 October 2019). Written informed consent was obtained from all participants.

### 2.3 *In vitro* fecal fermentation

A pH-regulated multichannel jar fermenter was employed to perform *in vitro* fecal fermentation, as described in a previous study (19). For metabolomic analysis, in total, 100 mL of YCFA medium was added to each vessel along with a carbon source. The carbon sources used were RD, Inu, and their mixtures. For the single carbon source groups, RD and Inu were added to the medium at a concentration of 1.0%. The mixture group (RD + Inu) was added to the medium at a concentration of 0.5% for each carbon source. For the control group, water was added to the medium instead of a carbon source. Before adding fecal samples, the pH of the medium was adjusted to 6.5. Fecal samples from eight donors were used individually without pooling and diluted to 10% (w/v) with saline. For each donor, 100 μL of the diluted fecal suspension was inoculated into each of four vessels corresponding to the control, RD, Inu, and RD + Inu conditions. Thus, each donor-derived fecal sample was tested under all four experimental conditions. This procedure was repeated for all eight fecal samples, resulting in a total of 32 fermentation reactions and eight reactions for each experimental condition. The fermentation was performed under anaerobic conditions with CO2 gas at 37 °C, with stirring at 200 rpm. The pH was maintained >6.0 with Na_2_CO_3_. For metabolomic analysis, after 24 h of fermentation, the cultured medium was collected and centrifuged at 8,000 × g for 3 min at 4 °C. To assess TMA production, 2 mM choline was added to the YCFA medium prior to fermentation. Following 24 and 48 h of fermentation, the culture medium was collected and centrifuged at 8,000 × g for 3 min at 4 °C. The culture supernatant was stored at −80 °C prior to metabolomic and TMA analyses. The culture sediment was also stored at −80 °C before microbiota composition analysis.

### 2.4 Microbiota analysis

Total DNA was extracted from the precipitate of fermentation medium using the bead-beating method. The V3–V4 region of the bacterial 16S rRNA gene was amplified and sequenced, as described in a previous study (23).

### 2.5 Metabolomic analysis

Metabolites were extracted from the fecal fermentation medium and analyzed using capillary electrophoresis-time-of-flight mass spectrometry (CE-TOFMS) and liquid chromatography time-of-flight mass spectrometry (LC-TOFMS) at Human Metabolome Technologies, Inc. (Tsuruoka, Yamagata, Japan), as previously described (21, 24, 25). Briefly, to analyze CE-TOFMS, cultured medium was centrifuged to remove debris, after which 80 μL of the supernatant was mixed with 20 μL of Milli-Q water containing internal standards (H3304-1002, Human Metabolome Technologies, Inc. (HMT), Tsuruoka, Yamagata, Japan). The mixture was centrifugally filtered through a 5-kDa cutoff filter (ULTRAFREE MC PLHCC, HMT) at 9,100 × g, 4 °C to remove macromolecules. The filtrate was then used for metabolomic analysis at HMT. An Agilent CE capillary electrophoresis system coupled with an Agilent 6,230 time-of-flight mass spectrometer (Agilent Technologies, Inc., Santa Clara, CA, USA) was used for CE-TOFMS. For LC-TOFMS analysis, the culture medium was centrifuged to remove debris, and 100 μL of the resulting supernatant was combined with 300 μL of methanol containing internal standards (H3304-1002). The mixture was centrifuged at 2,300 × g, 4 °C for 5 min. Following evaporation of the supernatant under a nitrogen stream, the residue was reconstituted in 100 μL of 50% isopropanol (v/v) for metabolomic analysis using LC-TOFMS at HMT. LC-TOFMS analysis was performed using an Agilent 1,260 HPLC system equipped with an Agilent 6,230 time-of-flight mass spectrometer (Agilent Technologies).

### 2.6 Quantitative analysis of trimethylamine

The concentration of TMA in the choline-added fermentation medium was analyzed using a liquid chromatography–tandem mass spectrometry system at Shimadzu Techno Research, Inc. (Kyoto, Japan). Serial dilutions of TMA (12.5–5,000 μmol/L) were prepared as standard samples, and calibration curves were created from these samples. In total, 10 μL samples were diluted with internal standard (0.5 mg/mL TMA-d9), 10 μL, and 480 μL 0.1% formic acid physiological saline solution/acetonitrile (1:1) mixture. The HPLC system consisted of a Shim-pack Velox HILIC column (150 mm × 2.1 mm internal diameter). The mobile phase consisted of (A) 50 mmol/L ammonium formate with 0.1% formic acid in water and (B) acetonitrile. The gradient programs for acetonitrile were 70% (0.0 min), 30% (4.0 min), 5% (5.0 min), and 70% (8.5 min). The flow rate was set to 0.25 mL/min. Mass spectrometry was performed using a Triple Quad 5,500 Plus system (AB Sciex Pte. Ltd., Tokyo, Japan).

### 2.7 Assessment of habitual diet and nutritional intake

Regular diet and nutrition intake were assessed using a brief self-administered diet history questionnaire (BDHQ) (26). The BDHQ consists of a four-page fixed-portion questionnaire that inquires about the intake frequency of selected food items to estimate the dietary intake of 58 foods and beverages over the preceding month (27). The participants read an explanation of the BDHQ and completed the questionnaire themselves, which was mailed to the Morinaga Milk Laboratory. After confirming that the questionnaire was complete, dietary habits and nutritional intake were assessed by Gender Medical Research, Inc. (Tokyo, Japan).

### 2.8 Statistical analysis

All statistical analyses were performed using Python (version 3.13). Differences in metabolomic profiles between the groups were evaluated by principal component analysis (PCA) and PERMANOVA following standardization (*μ* = 0, *σ* = 1). The paired Wilcoxon signed-rank test was used to compare metabolites and microbiota between the control and prebiotic groups of each participant. The Wilcoxon rank-sum test was used to compare microbiota and habitual diet between the TMA-high (>1.5 mM) and TMA-low (<1.5 mM) groups during choline-added medium fermentation. Correlations among metabolites, microbiota, and habitual diet were analyzed using Spearman’s rank correlation coefficient. Statistical significance was set at *p* < 0.05 using the Wilcoxon rank-sum test and Spearman’s rank correlation coefficient. This study was an exploratory analysis designed to identify harmful metabolites potentially reduced by prebiotics. Thus, no adjustment for the false discovery rate was performed in the metabolomics and microbiota analysis. However, the Holm–Bonferroni method was applied to adjust for multiple comparisons for each metabolite or bacterial taxon. The adjusted statistical significance levels were set at *p* = 0.0167, 0.025, and 0.05 for the first, second, and third comparisons, respectively.

## 3 Results

### 3.1 Microbiota composition

After 24 h of fermentation, microbiota composition was altered by the addition of prebiotics. The relative abundance of some beneficial bacteria, such as *Bifidobacterium_388775* and *Blautia_A_141781*, was higher in the prebiotic groups compared with the control group (Supplementary Figure S1).

### 3.2 Metabolites from resistant dextrin, inulin, and their combination

**Figure 1:**
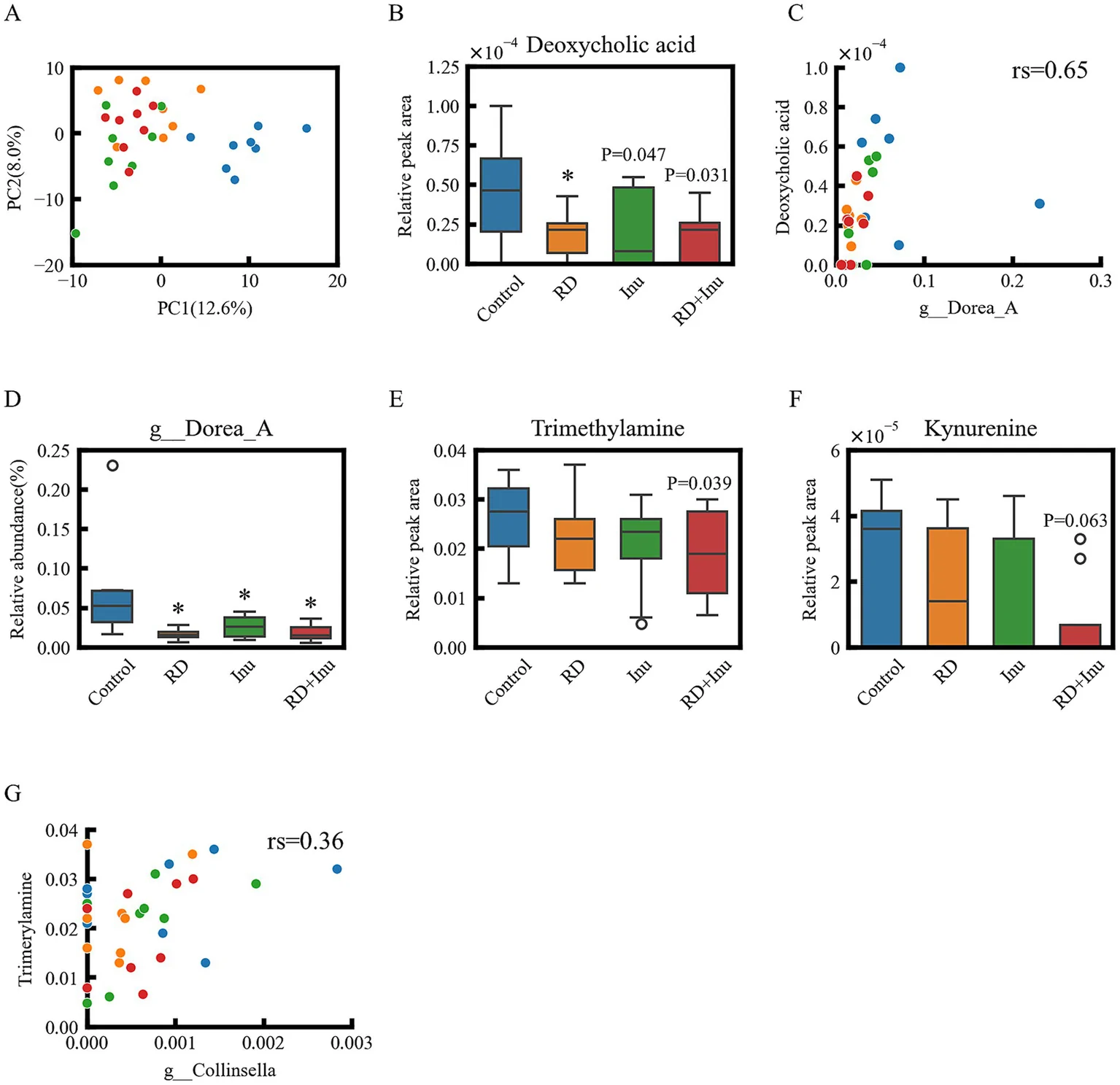
Metabolomic analysis from fecal fermentation after 24 h. Each experimental condition included eight donor-derived fermentation reactions (*n* = 8 per group). **(A)** Principal component analysis (PCA) of metabolites. **(B)** Relative peak area of deoxycholic acid in the fermentation medium. **(C)** Spearman’s correlation coefficient (*r*_s_) between *Dorea_A* and deoxycholic acid levels (*n* = 32). **(D)** Relative abundance of *Dorea_A*. **(E)** Relative peak area of trimethylamine (TMA) in the fermentation medium. **(F)** Relative peak area of kynurenine in the fermentation medium. **(B, D, E, F)** *p* values were calculated using the paired Wilcoxon signed-rank test and compared to the control group. Significant after Holm–Bonferroni multiple comparison adjustment. **(G)** Spearman’s correlation coefficient (*r*_s_) between *Collinsella* abundance and TMA levels (*n* = 32). **(A–G)** blue: control group, orange: RD group, green: Inu group, red: RD + Inu group.

PCA showed differences in the metabolites from fecal fermentation of older adults between the groups (Figure 1A). The control group and prebiotic groups (RD, Inu, and RD + Inu) were significantly different (*p* < 0.01, PERMANOVA). The RD and Inu were also significantly different (*p* < 0.01). No significant differences were observed between RD or Inu and RD + Inu. To analyze the changes in the metabolites by each single prebiotic group and their combination group, we compared each metabolite between the control and prebiotic groups. Prebiotic addition significantly altered various metabolites compared with the control group (Supplementary Tables S3A–C). Focusing on the harmful metabolites, the deoxycholic acid levels were significantly lower in the RD group than in the control group (Figure 1B). The RD + Inu group showed a decreasing trend in the deoxycholic acid levels (*p* = 0.031). To investigate the microbiota involved in deoxycholic acid production, we examined the correlation between deoxycholic acid levels and microbiota. A significant correlation was found between deoxycholic acid levels and *Dorea_A*, a deoxycholic acid-producing bacterium (rs = 0.65, *p* < 0.01, Figure 1C). *Dorea_A* was significantly lower in the prebiotic groups compared with the control group, and the reduction was particularly large in the groups containing RD (RD and RD + Inu) (Figure 1D). TMA and kynurenine were reduced in the RD + Inu group, although no statistically significant differences were found (*p* = 0.039 and *p* = 0.063, respectively; Figures 1E,F). A positive correlation was found between TMA levels and *Collinsella*, which harbor choline-TMA lyase (28) (rs = 0.36, *p* = 0.044, Figure 1G). No characteristic relationship was observed between kynurenine and kynurenine-producing bacteria (data not shown).

### 3.3 Reducing TMA production by the combination of resistant dextrin and inulin

**Figure 2:**
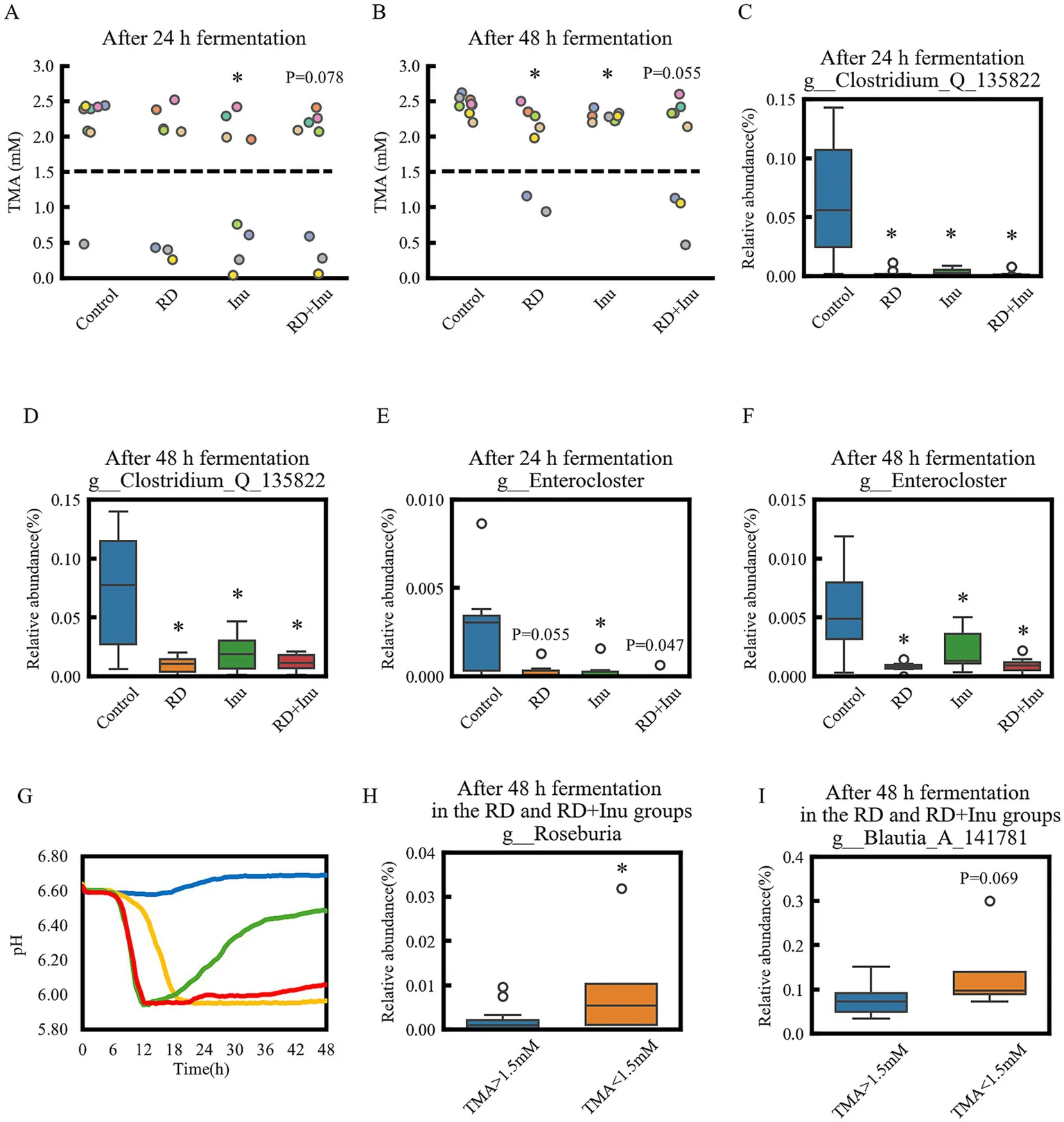
In total, 24 h and 48 h of fecal fermentation in choline-supplemented medium. Each experimental condition included eight donor-derived fermentation reactions (*n* = 8 per group). **(A)** The trimethylamine (TMA) concentration after 24 h of fermentation. The color of the circle indicates the data for each sample. **(B)** The TMA concentration after 48 h of fermentation. **(C)** Relative abundance of *Clostridium_Q_135822* after 24 h of fermentation. **(D)** Relative abundance of *Clostridium_Q_135822* after 48 h of fermentation. **(E)** Relative abundance of *Enterocloster* after 24 h of fermentation. **(F)** Relative abundance of *Enterocloster* after 48 h of fermentation. **(A–F)** *p* values were calculated using the paired Wilcoxon signed-rank test and compared with the control group. ^*^Significant after Holm–Bonferroni multiple comparison adjustment. **(G)** pH of fermentation medium after 48 h of fermentation. Blue: Control group, orange: RD group, green: Inu group, red: RD + Inu group. **(H)** Relative abundance of *Roseburia* in the RD and RD + Inu groups after 48 h of fermentation at high TMA (>1.5 mM, *n* = 11) and low TMA (<1.5 mM, *n* = 5). **(I)** Relative abundance of *Blautia_A_141781* in the RD and RD + Inu groups after 48 h of fermentation at TMA > 1.5 mM (*n* = 11) and TMA < 1.5 mM (*n* = 5). **(H, I)** *p < 0.05 Wilcoxon rank-sum test.

Among the harmful metabolites reduced in the RD + Inu group, we focused on the TMA because its metabolite TMA-N-oxide is associated with some diseases, such as cardiovascular and kidney diseases (29, 30). TMA is produced from choline and carnitine by the gut microbiota. Referring to a previous fecal fermentation study (10), we added choline or carnitine to the medium and conducted long-term fecal fermentation (48 h) to reevaluate the reduction of TMA production by RD, Inu, and their combination. The addition of choline increased TMA production, whereas carnitine did not (Supplementary Table S4). Therefore, we selected a choline-supplemented medium to evaluate the effects of TMA inhibition. After 24 h of fermentation, TMA levels were significantly lower in the Inu group compared with the control group (Figure 2A). After 48 h of fermentation, TMA levels were significantly reduced in the RD and Inu groups, and a decreasing trend was observed in the RD + Inu compared with the control group (*p* = 0.055, Figure 2B). The effects of prebiotics on TMA production widely varied among individuals. After 24 h of fermentation, TMA levels were highly reduced (<1.5 mM) in three fecal samples from the RD group, four from the Inu group, and three from the RD + Inu group. After 48 h of fermentation, there were no fecal samples with highly reduced TMA levels in the Inu group, two in the RD group, and three in the RD + Inu group. Analysis of the microbiota in the fermentation medium showed that *Clostridium_Q_135822*, which harbor choline-TMA lyase (28), was significantly lower in the prebiotic group compared with the control group after 24 h and 48 h of fermentation (Figures 2C,D). *Enterocloster*, which also harbors a TMA-producing enzyme (31), was significantly decreased in the Inu group after 24 h of fermentation and in the prebiotic groups after 48 h of fermentation compared with the control group (Figures 2E,F). The relative abundances of *Clostridium_Q_135822* and *Enterocloster* after 48 h of fermentation were higher than those after 24 h of fermentation, especially in the Inu group. The pH of the fermentation medium decreased to 6.0 after 12 h of fermentation in the Inu group and then increased (Figure 2G). The RD group showed a gradual decrease in the pH of the medium compared with that of the Inu group and maintained a low pH after 48 h of fermentation. RD + Inu lowered the pH after 12 h of fermentation and maintained a low pH during 48 h of fermentation. There was no significant difference in pH between samples with highly reduced TMA levels (<1.5 mM) and those with less reduction (>1.5 mM) in the RD and RD + Inu groups after 48 h of fermentation (*p* = 0.87). In the same groups and at the same time point, *Roseburia* was significantly lower, and *Blautia_A_141781* tended to decrease (*p* = 0.069) in the highly reduced TMA group (Figures 2H,I).

### 3.4 Comparison of fecal samples with low TMA concentrations to other samples

**Figure 3:**
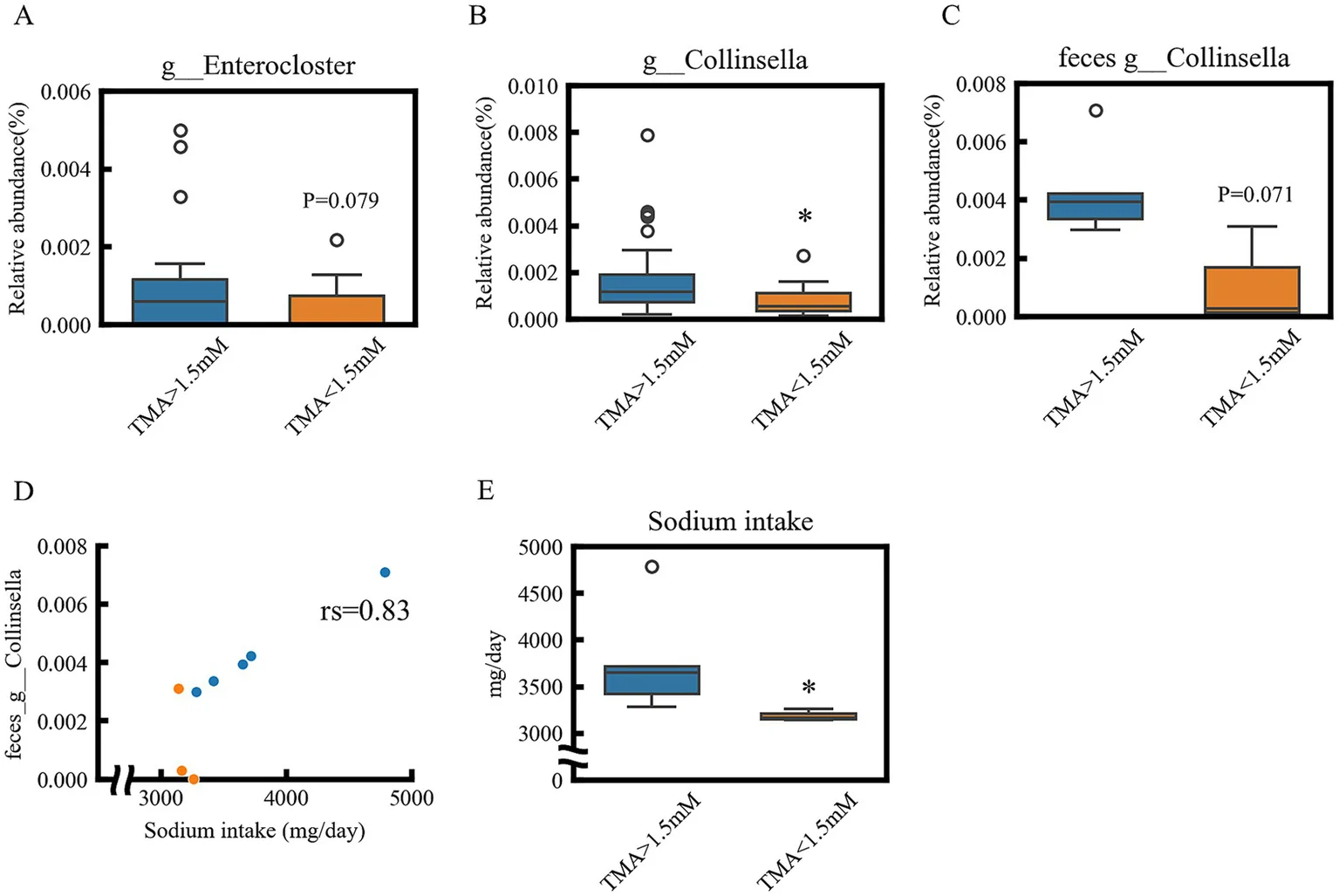
Comparison of groups with high trimethylamine (TMA) (>1.5 mM) and low TMA (<1.5 mM) after 24 h and 48 h of fermentation with choline-added medium. **(A)** Relative abundance of *Enterocloster* in the fermentation medium of the TMA < 1.5 mM (*n* = 15) and TMA > 1.5 mM (*n* = 33) groups. **(B)** Relative abundance of *Collinsella* in the fermentation medium of the TMA < 1.5 mM and TMA > 1.5 mM groups. **(C)** Relative abundance of *Collinsella* in the feces of the TMA < 1.5 mM (*n* = 3) and TMA > 1.5 mM groups (*n* = 5). **(D)** Spearman’s correlation coefficient (*rs*) between sodium intake and relative abundance of *Collinsella* in the feces (*n* = 8). Blue: TMA > 1.5 mM group, orange: TMA < 1.5 mM group. **(E)** Sodium intake of participants in the TMA > 1.5 mM group (*n* = 5) and the TMA < 1.5 mM group (*n* = 3). **(A–C, E)** ^*^p < 0.05 Wilcoxon rank-sum test.

To investigate the mechanism of individual differences in the reducing effect of TMA by prebiotic addition, we compared the microbiota composition in the fermentation medium with low TMA production (<1.5 mM) to that in the other samples among the prebiotic groups (three RD, four Inu, and three RD + Inu after 24 h of fermentation, two RD and three RD + Inu after 48 h of fermentation vs. the remaining 24 + 48 h of fermentation in the prebiotic groups). *Enterocloster* showed a lower trend in the TMA < 1.5 mM group than in the TMA > 1.5 mM group (*p* = 0.079, Figure 3A). *Collinsella* was significantly lower in the TMA < 1.5 mM group than in the TMA > 1.5 mM group (Figure 3B). To reveal the features of the fecal samples that highly reduced TMA with the combination of RD and Inu, we compared the microbiota in the feces of the three samples that showed low TMA concentrations (<1.5 mM) in the RD + Inu group after 48 h of fermentation and the other five samples. In the group with TMA levels < 1.5 mM, *Collinsella* tended to be lower than in the group with TMA levels > 1.5 mM (*p* = 0.071, Figure 3C). Analysis of the correlation between *Collinsella* and habitual nutritional intake of fecal donors showed that sodium intake was positively correlated with *Collinsella* (rs = 0.83, *p* = 0.010, Figure 3D). Sodium intake was significantly lower in the TMA < 1.5 mM group than in the TMA > 1.5 mM group (Figure 3E). A positive correlation trend was observed between *Collinsella* and the habitual intake of red meats, which contain choline and carnitine (rs = 0.64, *p* = 0.086, data not shown).

## 4 Discussion

This study showed that the combination of RD and Inu could reduce harmful metabolites, such as deoxycholic acid, kynurenine, and TMA, in *in vitro* fecal fermentation using samples from older adults. Although several of these reductions did not reach statistical significance, these findings provide preliminary evidence that this prebiotic combination may inhibit harmful metabolite production that should be reduced in older adults.

Deoxycholic acid, which is produced from bile acid by the gut microbiota, disrupts intestinal barrier function and is involved in the development of colon cancer (32, 33). In this study, deoxycholic acid level was significantly lower in the RD group compared with that in the control group. RD also reduced fecal deoxycholic acid levels in a previous human study, suggesting that the findings of this *in vitro* fecal fermentation study reflect those observed in the clinical study (34). The underlying mechanism is thought to involve the stabilization of the micellar structure of bile acids by RD, which reduces their availability as a source for deoxycholic acid production and consequently lowers deoxycholic acid levels (35). However, this was an *in vitro* fecal fermentation study; therefore, the reduced deoxycholic acid production may not have been caused by the stabilization of the micellar structure of the bile acids. In this study, *Dorea_A*, a deoxycholic acid-producing bacterium (36, 37), was reduced in the prebiotic groups, specifically in the RD group. This finding suggests a possible association between reduced *Dorea_A* abundance and decreased deoxycholic acid production by RD. However, the observed correlation between *Dorea_A* abundance and deoxycholic acid levels does not establish causation. Future functional or metagenomic studies are needed to confirm the underlying mechanism. In addition, deoxycholic acid levels showed a decreasing trend in the RD + Inu group, which contained a smaller amount of RD than the RD group, suggesting that combining RD with Inu may be effective, even when the amount of RD is reduced.

Although the causal relationship between kynurenine, a tryptophan metabolite, and depression remains unclear, some studies have suggested that elevated kynurenine levels are associated with depression (38–40). Kynurenine is produced not only by host metabolism but also by the gut microbiota. It has been studied as a substance involved in the “gut–brain axis,” in which the gut microbiota influences brain function (41). Few studies have examined the effects of prebiotic intake on kynurenine production. For example, Inu intake does not reduce blood kynurenine levels in obese women (42). In contrast, intake of RD reduces blood kynurenine levels in patients with type 2 diabetes (43). In this study, the RD and Inu combination group showed a decreasing trend in kynurenine levels although the difference was not statistically significant. This suggests that the combination of RD and Inu could reduce kynurenine production. Further studies are required to confirm whether this combination suppresses kynurenine production.

The gut microbiota converts choline and carnitine into TMA, which is elevated in the microbiota of older adults and impairs intestinal barrier function (15, 44). In addition, TMA is converted to TMA-N-oxide in the liver. TMA-N-oxide modulates platelet hyperresponsiveness and enhances thrombosis risk (45). Increased plasma TMA-N-oxide levels are associated with increased risk of cardiovascular and kidney diseases. Therefore, it is important to reduce TMA production in older adults, who have a higher risk of developing various diseases than younger people. In this study, Inu inhibited the production of TMA during fecal fermentation in choline-supplemented medium. However, although the number of TMA-low samples (<1.5 mM) was four after 24 h in the Inu group, all samples showed increased TMA, and there were no TMA-low samples after 48 h of fermentation. Furthermore, TMA-producing bacteria increased from 24 h to 48 h of fermentation in the Inu group. Therefore, it was hypothesized that Inu would not inhibit TMA production for an extended period. In a previous study, Inu was utilized faster than the RD by the gut microbiota and could not maintain a prebiotic effect for a long time (19). In addition, some human studies have shown that Inu intake does not reduce plasma TMA-N-oxide levels (46, 47). Therefore, it was suggested that Inu could inhibit TMA production, but could not be maintained for a long time, and the inhibitory effect was limited by Inu alone. In contrast, TMA-low samples were observed in the RD and RD + Inu groups after 48 h of fermentation. It was reported that RD inhibited TMA production more than other prebiotic materials, such as fructooligosaccharides and maltooligosaccharides (48). A human study also showed that RD intake reduced fecal TMA production in participants with high baseline TMA levels (34). Moreover, in this study, the number of TMA-low samples was the highest in the RD + Inu group after 48 h of fermentation. A fecal fermentation study assessing TMA production showed that when the pH of the fermentation medium was not controlled, the pH of the medium decreased and TMA was not produced (10). In humans, TMA-producing bacteria increase in the distal colon, which prebiotics cannot reach, and the pH is higher than that in the proximal colon (11). Therefore, a lower pH is important for inhibiting TMA production. RD is utilized more slowly by the gut microbiota than Inu and maintains a lower pH during long-term fermentation (19, 49). In the present study, the combination of RD and Inu decreased the pH after 12 h of fermentation and maintained a lower pH during fermentation. The number of low TMA samples was the largest after 48 h of fermentation in the RD + Inu group. However, TMA-high samples also showed a lower pH in the combination group, and there may be other reasons for the inhibition of TMA production. Comparison between the TMA > 1.5 mM group and the TMA < 1.5 mM group in the RD and RD + Inu groups after 48 h of fermentation showed that the abundance of certain beneficial bacteria (*Roseburia* and *Blautia_A_141781* (50, 51)) was higher in the TMA < 1.5 mM group than in the TMA > 1.5 mM group. These results suggest that the improvement in gut microbiota is more pronounced in the TMA < 1.5 mM group, which was associated with greater suppression of TMA production. Moreover, *Blautia* decreased in the older-adult-type microbiota, and TMA was higher in the feces than in the adult-type microbiota and feces (15). Therefore, even with the same prebiotic material, changes in the microbiota may differ depending on the fecal sample, thereby influencing the inhibitory effects of TMA. Further functional or metagenomic analyses are necessary to determine whether these microbiota changes directly contribute to inhibiting TMA production in the RD + Inu group.

Individual responses to prebiotics depend on the composition of the gut microbiota and have been studied to reveal the characteristics of prebiotic responders (52). Individual dietary habits influence responses to prebiotics (53, 54). In this study, participants who provided fecal samples with low TMA levels in the RD + Inu group after 48 h of fermentation had a lower sodium intake than the other participants, and a positive correlation was found between sodium intake and *Collinsella*, which harbors a TMA-producing enzyme (28). Changes in the microbiota induced by sodium intake have been studied (55), but few studies have investigated TMA-producing bacteria. An animal study assessing the relationship between sodium intake and TMA production showed that a high-salt diet increased TMAO in the blood, but did not increase TMA production or TMA-producing bacteria in the feces (56). Although the present study did not measure TMAO in the blood or TMA in the feces and could not directly compare with a previous animal study, it suggests that sodium intake affects the abundance of TMA-producing bacteria and the response to TMA inhibition by prebiotics. Sodium intake is associated with the development of cardiovascular diseases (57). A recent study suggested that changes in the gut microbiota induced by sodium intake might also be related to cardiovascular diseases (58). Based on these results, it is suggested that sodium intake reduces the inhibitory effect of prebiotics on TMA production, which may also be involved in cardiovascular diseases. Further studies are necessary to elucidate the influence of sodium intake on the inhibitory effects of prebiotics on TMA production.

This study has some limitations. The main limitation of this study is its small sample size. Although this study revealed the relationship between individual diets and the prebiotic TMA-inhibition effect, the sample size was significantly small to adequately assess the individual response to prebiotics. To elucidate the relationship between a habitual diet and the inhibition of TMA production by prebiotics, a larger sample size is necessary. In addition, false discovery rate correction was not applied in the metabolomics analysis to increase statistical power and avoid overlooking potentially relevant metabolites; however, this approach may have increased the risk of false-positive findings. Therefore, results should be interpreted as exploratory and hypothesis-generating. Second, this study was conducted in healthy Japanese older adults. Because participants were not maintained under standardized dietary conditions, the effects of dietary background on harmful metabolite production and the efficacy of prebiotics could not be fully evaluated. In addition, the generalizability of these findings to populations with different dietary habits or demographic characteristics remains uncertain. Future studies under standardized dietary conditions and in more diverse populations are warranted. Third, we analyzed TMA levels in the fecal fermentation medium after 24 h and 48 h, and TMA production reached a plateau at 24 h in most samples. Assessing the TMA before 24 h of fermentation may reveal the mechanism underlying the inhibitory effect of prebiotics on TMA production. Finally, this was an *in vitro* fecal fermentation study, which cannot fully reproduce the human intestinal environment, including physiological substrate concentrations and individual differences in anaerobic conditions. Moreover, this model does not account for host physiological interactions, such as barrier function, immune responses, absorption, metabolism, and intestinal transit. Therefore, it remains unclear whether the observed metabolite changes would also occur in humans. Clinical studies involving older adults are necessary to verify these results.

In conclusion, the combination of RD and Inu could reduce certain harmful metabolites produced by the gut microbiota in older adults. These preliminary results may contribute to more effective strategies for reducing disease risk and to the development of food products containing prebiotics for older adults. To confirm these findings, additional studies with larger numbers of fecal samples and clinical trials are necessary.

## Data Availability

The data presented in this study can be found in the article and its Supplementary material. Further enquiries can be directed to the corresponding author.
