# Supplementary material for: Combined effects of resistant dextrin and inulin on the reduction of harmful metabolites during in vitro fecal fermentation in older adults
Source: Front Nutr. 2026 Sep 3;13:1909212. doi: 10.3389/fnut.2026.1909212 (PMC13584765; doi:10.3389/fnut.2026.1909212)
Supplement: Supplementary file 1 [file Supplementary_file_1.docx]

Supplementary Material

## 1.1 Supplementary Figures

**Supplementary Figure S1 Bacterial population after 24 h of fermentation.** Top 30 predominant bacterial genera in the fecal fermentation samples. Colors indicate the percentage of each bacterial population.
